# Supplementary material for: Anomalous Landau quantization in intrinsic magnetic topological insulators
Source: Nat Commun. 2023 Aug 9;14:4805. doi: 10.1038/s41467-023-40383-x (PMC10412595; doi:10.1038/s41467-023-40383-x)
Supplement: Supplementary file 1 — Supplementary Information [file 41467_2023_40383_MOESM1_ESM.pdf]

Supplementary Information

Anomalous Landau quantization in intrinsic magnetic topological insulators

Su Kong Chong[1†]*, Chao Lei[2†], Seng Huat Lee[3,4], Jan Jaroszynski[5], Zhiqiang Mao[3,4], Allan H. MacDonald[2], Kang L. Wang[1]*

[1]Department of Electrical and Computer Engineering, University of California, Los Angeles, California 90095, United States

[2]Department of Physics, The University of Texas at Austin, Austin, TX 78712

[3]2D Crystal Consortium, Materials Research Institute, The Pennsylvania State University, University Park, PA 16802, USA

[4]Department of Physics, The Pennsylvania State University, University Park, PA 16802, USA

[5]National High Magnetic Field Laboratory, Florida State University, Tallahassee, FL, USA

*Corresponding authors: sukongc@g.ucla.edu (S.K.C.); wang@seas.ucla.edu (K.L.W.)

†These authors contributed equally to this work

Contents:

Supplementary Note 1. Theoretical models

Supplementary Note 2. Additional transport data

Supplementary Note 1

Theoretical models

The coupled Dirac cone model considers only the Dirac cone surface states on the top and bottom surfaces of each septuple layer and hoppings between these Dirac cones [1]. For each Dirac cone, there is an exchange splitting contributed by the magnetic moments of Mn atoms. With these ingredients, the Hamiltonian reads as Supplementary Equation 1:

$$H = \sum_{k_\perp, ij}\left[\left((-)^i \hbar v_D (\hat{z} \times \boldsymbol{\sigma}) \cdot \mathbf{k}_\perp + m_i\, \sigma_z\right)\delta_{ij} + \Delta_{ij}(1-\delta_{ij})\right] c^\dagger_{k_\perp i} c_{k_\perp j},$$

where i and j label the Dirac cone, $\hbar$ is the reduced Planck constant, v_D is the velocity of the Dirac cones, and $\boldsymbol{\sigma}$ is the vector of Pauli matrices. $m_i = \sum_\alpha M_\alpha J_\alpha$ is the exchange splitting of the ith Dirac cone with $M_\alpha = \pm 1$ the direction of magnetic moments from the Mn atoms of the α layer. The hopping between the ith and jth Dirac cones is denoted by Δ_{ij}. Here we only retain the most dominant parameters, i.e., the hopping between two Dirac cones within the same layer Δ_S and between two adjacent layers Δ_D, the exchange splittings only consider the contributions from the magnetic moments of Mn atoms in the same layer J_S and from adjacent layers J_D. These parameters in the model can be estimated by comparing the band energies at Γ and Z points from the couple Dirac cone model and DFT calculations.

DFT calculations were performed using Vienna Ab initio Simulation Package (VASP) [2] in which generalized gradient approximations (GGA) of Perdew-Burke-Ernzerhof (PBE) [3] have been adopted for exchange-correlation potential. On-site correlation on the Mn-3d states is treated by performing a simplified (rotationally invariant) approach to the LSDA+U calculations [4] with $U - J$ equals 5.34 eV. Here U and J represent the strength of the effective on-site Coulomb and

exchange interactions. Supercells that correspond to $2 \times 2 \times 1$ of the original primitive cells of $MnBi_2Te_4$ were used to simulate the Sb doping in the bulk material with ferromagnetic spin configuration. The global break condition E_{diff}, i.e. the relaxation of the electronic degrees of freedom will be stopped if the total (free) energy change and the band structure energy change ('change of eigenvalues') between two steps are both smaller than E_{diff}, for the electronic self-consistency loop is set to be 10^{-6} eV, and a 9 × 9 × 3 Γ-centered k-point integration grid was employed.

In the presence of a perpendicular external magnetic field $\mathbf{B} = B\hat{z}$ and choose the Landau gauge $\mathbf{A} = (0, -Bx, 0)$, the quantized Dirac Hamiltonian becomes as Supplementary Equation 2:

$$H_D = \frac{1}{2}\hbar\omega_c(\sigma^+ a^\dagger + \sigma^- a)$$

where $\sigma^\pm = \sigma_x \pm \sigma_y$, $a = 1/\sqrt{2}(\tilde{x} + \partial\tilde{x})$, $a^\dagger = 1/\sqrt{2}(\tilde{x} - \partial\tilde{x})$ and $\omega_c = \hbar v_D/\sqrt{2}l_B$. Here $l_B = \sqrt{hc/eB}$ is the magnetic length, $\tilde{x} = l_B k - x/l_B$ and $\partial\tilde{x} = l_B\partial_x$. The general wavefunction in Landau level representation is then expressed as Supplementary Equation 3:

$$|n, i\sigma\rangle = \sum\nolimits_j \big(C_{nj\uparrow}|n, j\uparrow\rangle + C_{nj\downarrow}\,|n-1, j\downarrow\rangle\big)$$

with i,j represents the surface indexes, σ =↑/↓ labels the spin, and n = 0, 1,… is the Landau level index. $C_{nj}\uparrow$ is the coefficient of the nth basis Landau level function. For n = 0 Landau levels, the wavefunctions are spin-polarized, and the Hamiltonian in n = 0 Landau level subspace is reduced to a generalized Su–Schrieffer–Heeger (SSH) model which is independent of the magnetic field.

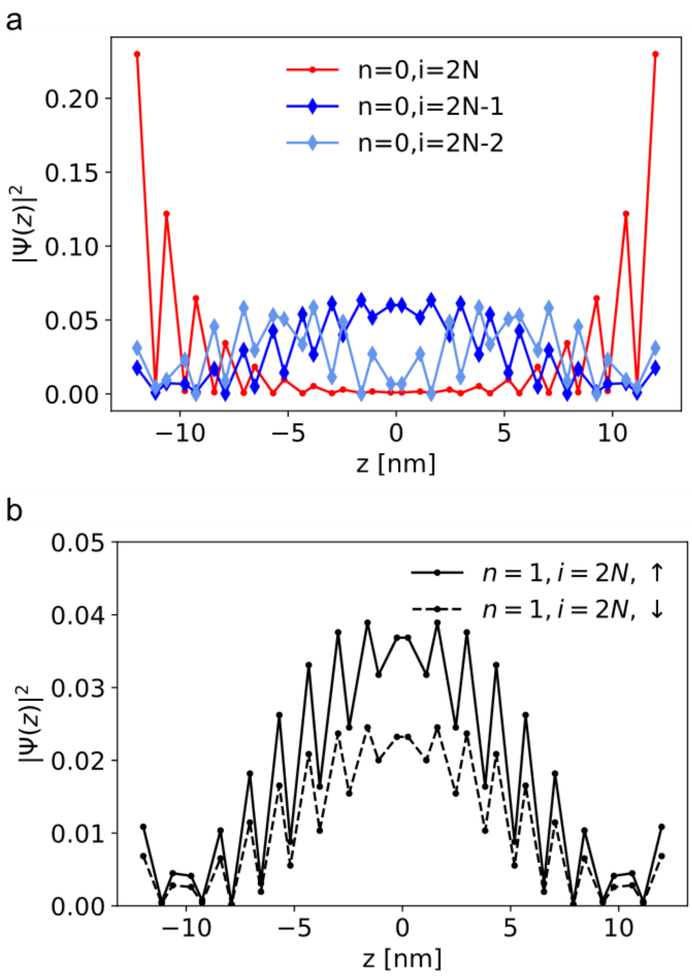

Supplementary Figure 1. Wavefunction distribution of selected Landau band indices, i, calculated from Supplementary Equation 3 for the (a) n = 0 anomalous LLs, and (b) n = 1 non-anomalous LLs for an 18SL MBST. Here z is the position along the out-of-plane direction and N is the number of septuple layers. The n = 0 anomalous LLs are spin-polarized, while the solid and dashed lines in (b) represent the spin up and down components for non-anomalous LLs. The wavefunctions of the n = 0 anomalous LLs shift gradually from surface to bulk with the increment of i.

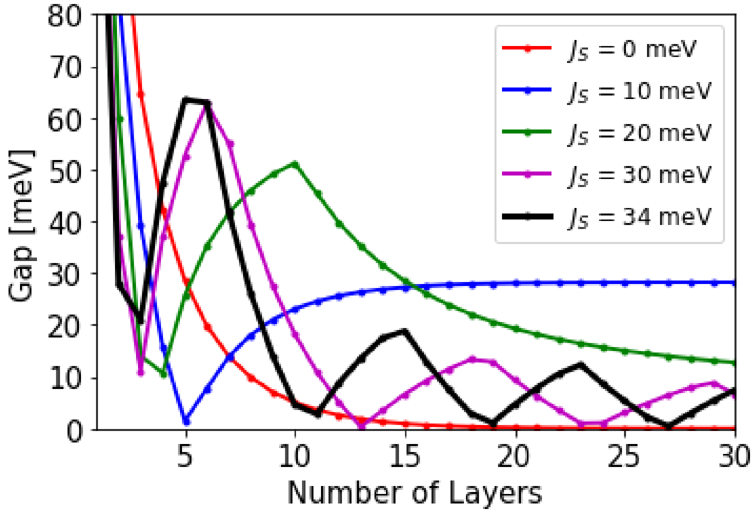

Supplementary Figure 2. Calculated magnetic gap size as a function of thickness for ferromagnetic $Mn(Bi_{1-x}Sb_x)_2Te_4$ thin films with 25% Sb doping level at different intralayer exchange splitting (J_S). The value of exchange splitting may vary for different samples depending on the antisite defects, disorder etc. Topological phase transition happens when the gap closes and the Chern number increases by one every time the gap closes.

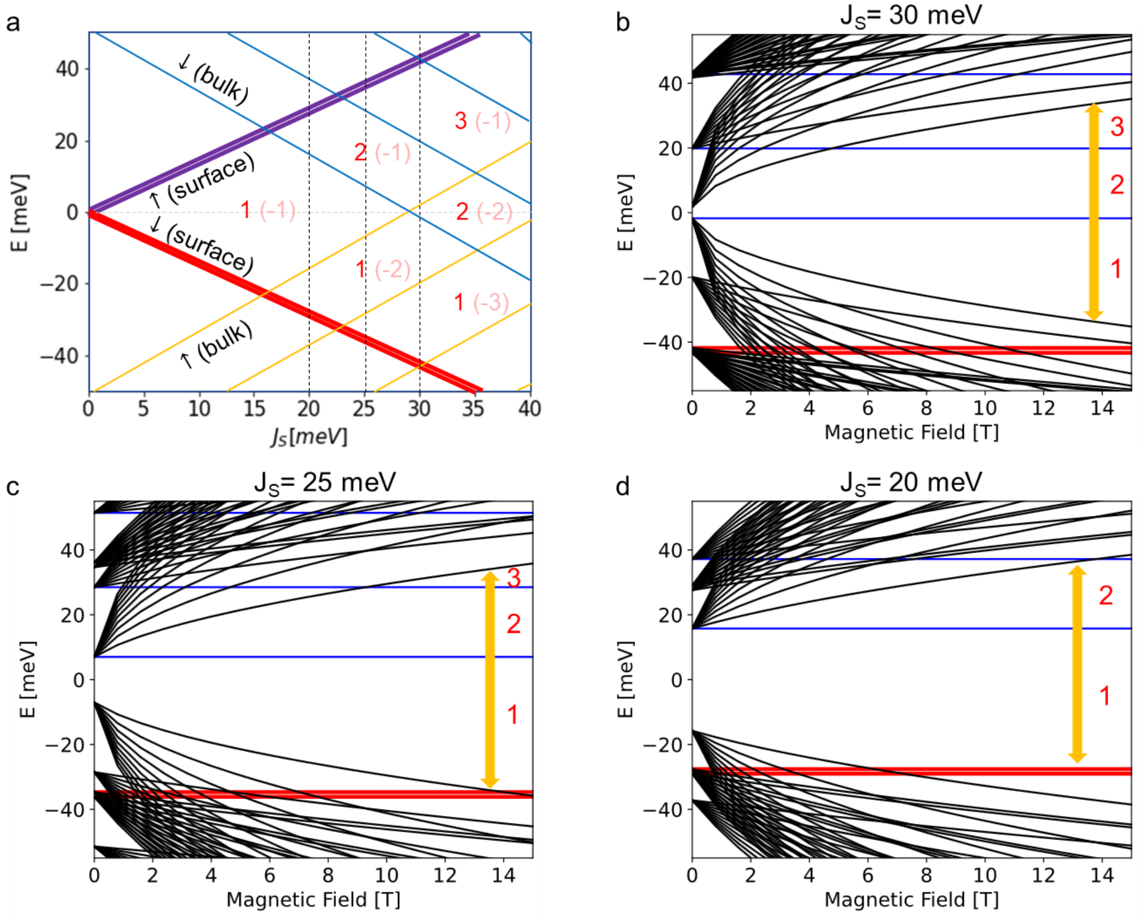

Supplementary Figure 3. Landau level fan diagrams and filling factors of a 14-SL $Mn(Bi_{0.75}Sb_{0.25})_2Te_4$ film. (a) Band energies at 2D wavevector $\mathbf{k} = 0$ at zero magnetic field versus the same-layer exchange splitting, J_S. The bold green (red) curve is for the spin up (down) state localized at the thin film surfaces, while the orange (blue) curve denotes the spin up (down) states distributed in the bulk. The Chern numbers of the anomalous LLs for down (up)-spins are indexed in (a). (c) and (d) Landau level structures for thin film with an aligned moment spin configuration at J_S of (b) 30, (c) 25, and (d) 20 meV, as labeled in (b) with black vertical lines. The $n \neq 0$ non-anomalous Landau levels are plotted with black curves, while the $n = 0$ anomalous Landau levels whose energies are independent of magnetic field are plotted with blue and red curves. The red (blue) curves distinguish the anomalous Landau levels that are localized at the surface (in the bulk).

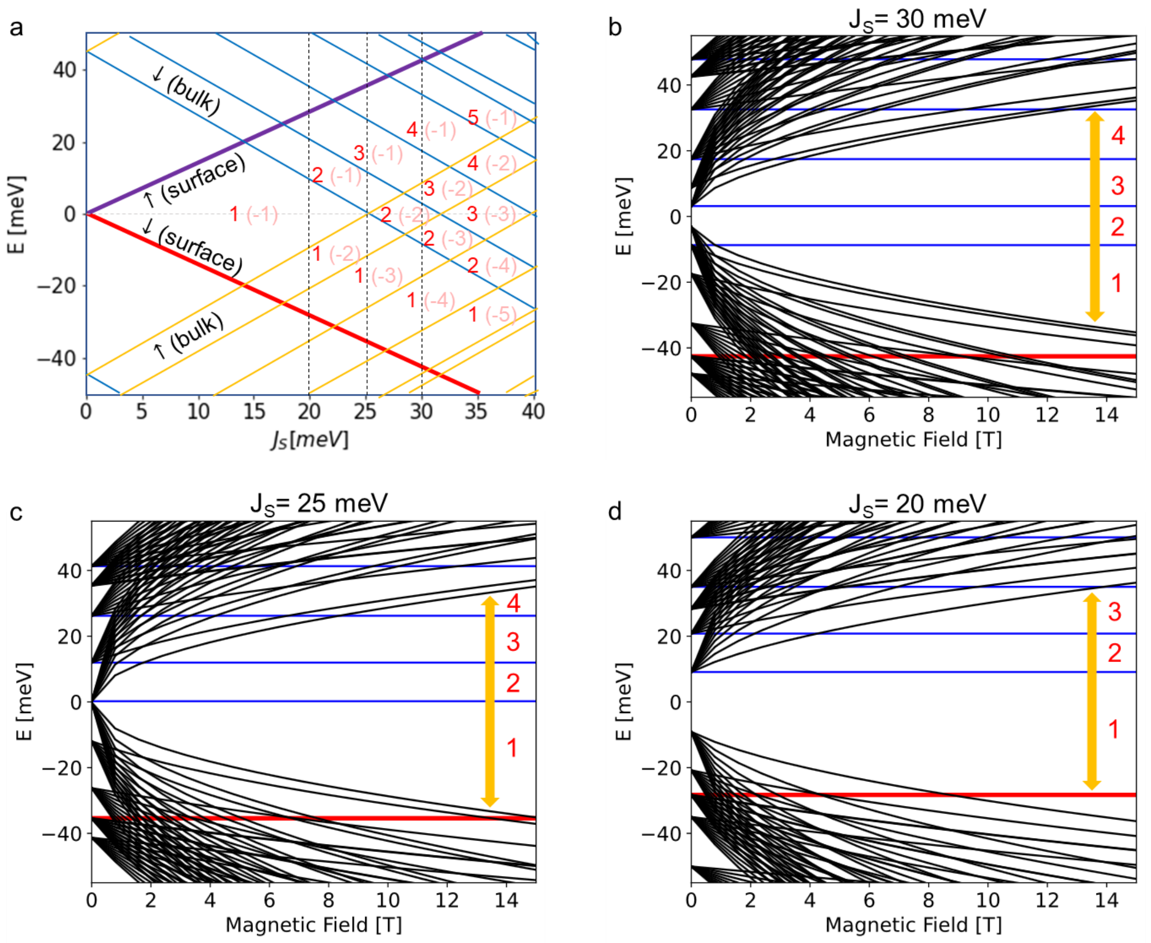

Supplementary Figure 4. Landau level fan diagrams and filling factors of a 21-SL $Mn(Bi_{0.75}Sb_{0.25})_2Te_4$ film. (a) Band energies at 2D wavevector $\mathbf{k} = 0$ at zero magnetic field versus the same-layer exchange splitting, J_S. The bold green (red) curve is for the spin up (down) state localized at the thin film surfaces, while the orange (blue) curve denotes the spin up (down) states distributed in the bulk. The Chern numbers of the anomalous LLs for down (up)-spins are indexed in (a). (c) and (d) Landau level structures for the thin film with an aligned moment spin configuration at J_S of (b) 30, (c) 25, and (d) 20 meV, as labeled in (b) with black vertical lines. The n $\neq$ 0 non-anomalous Landau levels are plotted with black curves, while the n = 0 anomalous Landau levels whose energies are independent of magnetic field are plotted with blue and red curves. The red (blue) curves distinguish the anomalous Landau levels that are localized at the surface (in the bulk).

Supplementary Note 2

Additional transport data

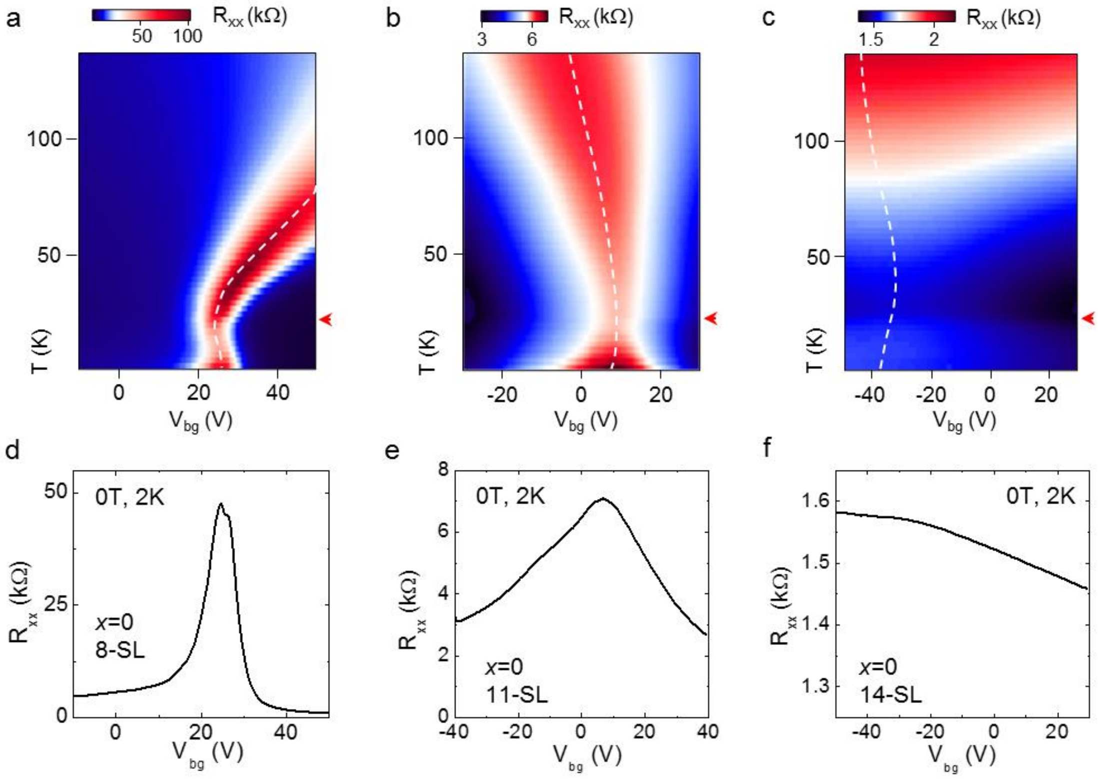

Supplementary Figure 5. Temperature-dependent transport for $MnBi_2Te_4$. Color maps of R_{xx} as functions of temperature and gate voltage for $MnBi_2Te_4$ at flake thickness of (a) 8-SL, (b) 11-SL, and (c) 14-SL. White dashed lines trace the shift of charge neutrality point with temperature. Red arrows point to the cusp at T_N for the paramagnetic to AFM transition. Gate-dependent R_{xx} measured at zero magnetic field and temperature of 2K for the (d) 8-SL, (e) 11-SL, and (f) 14-SL $MnBi_2Te_4$.

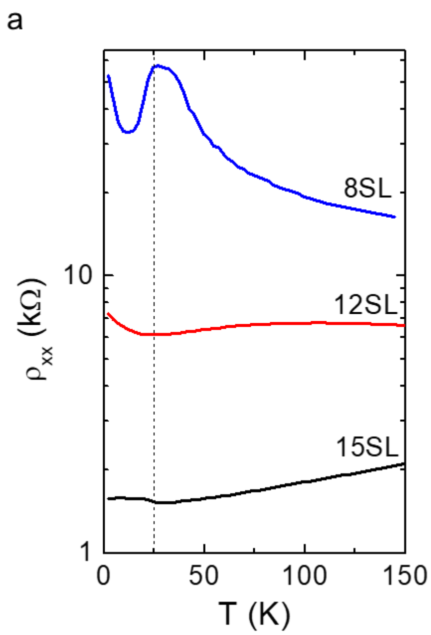

Supplementary Figure 6. Resistivity-temperature response. The line profiles of ρ_{xx} versus temperature for (a) $MnBi_2Te_4$, and (b) $Mn(Bi_{0.74}Sb_{0.26})_2Te_4$ at different flake thicknesses. The cusp at the T_N of ~25K and ~24K for $MnBi_2Te_4$ and $Mn(Bi_{0.74}Sb_{0.26})_2Te_4$ are indicated by black dashed lines. The surface-like and bulk-like behaviors can be distinguished from the insulating and metallic temperature responses as revealed in the 8-SL and 15-SL $MnBi_2Te_4$, respectively. Whereas for the $Mn(Bi_{0.74}Sb_{0.26})_2Te_4$, the surface-like behavior can be observed up to 21-SL.

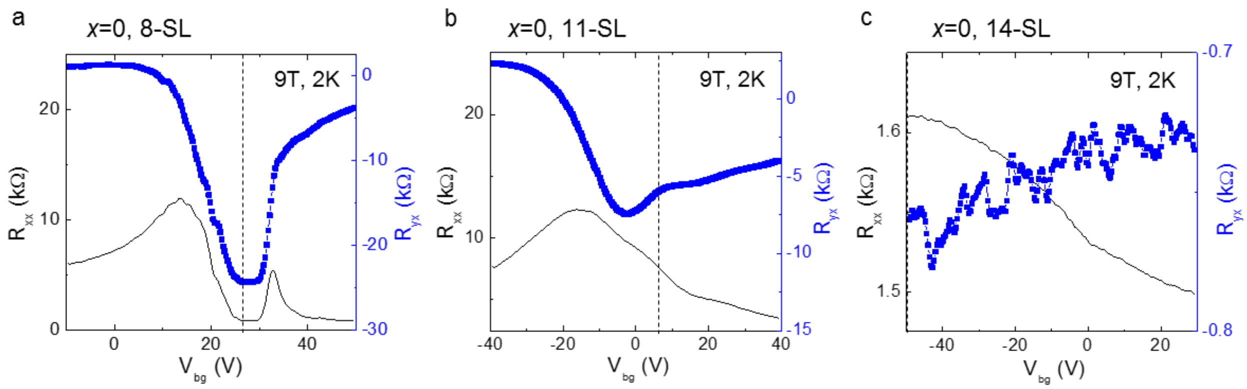

Supplementary Figure 7. Magnetotransport for $MnBi_2Te_4$. Gate dependent R_{xx} (black lines) and R_{yx} (blue dots) for the (a) 8-SL, (b) 11-SL, and (c) 14-SL $MnBi_2Te_4$ measured at magnetic field of 9T and temperature of 2K. The vertical dashed lines indicate the backgate voltages corresponding to the ρ_{xx}^{CNP} at zero magnetic field.

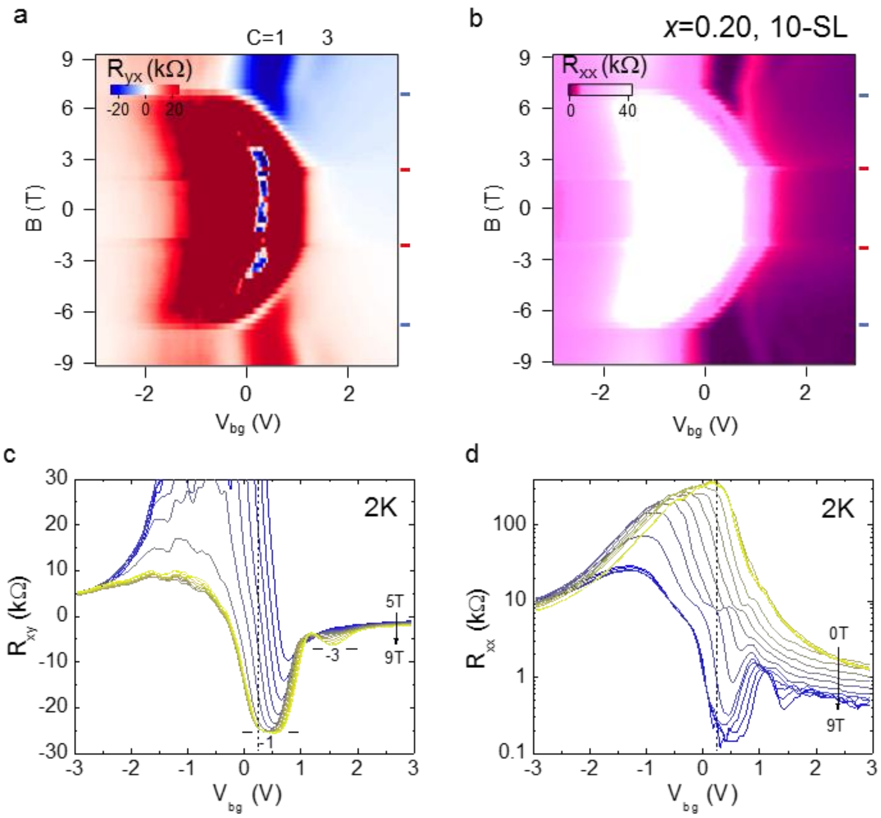

Supplementary Figure 8. Magnetotransport for 10-SL $Mn(Bi_{0.8}Sb_{0.2})_2Te_4$. Color maps of (a) R_{yx}, and (b) R_{xx} as functions of magnetic field and gate voltage for the 10-SL $Mn(Bi_{0.8}Sb_{0.2})_2Te_4$ measured at a temperature of 2K. The spin-flop and spin-flip fields are indicated by the red and blue lines, respectively. The C = 1 and 3 states are indexed in (a). Plots of (c) R_{yx} and (d) R_{xx} versus backgate voltage at different magnetic fields for the 10-SL $Mn(Bi_{0.8}Sb_{0.2})_2Te_4$. The C = 1 and 3 states are indexed in R_{yx} plots in (c). The vertical dashed lines in (c) and (d) indicate the backgate voltage corresponding to the ρ_{xx}^{CNP} at zero magnetic field.

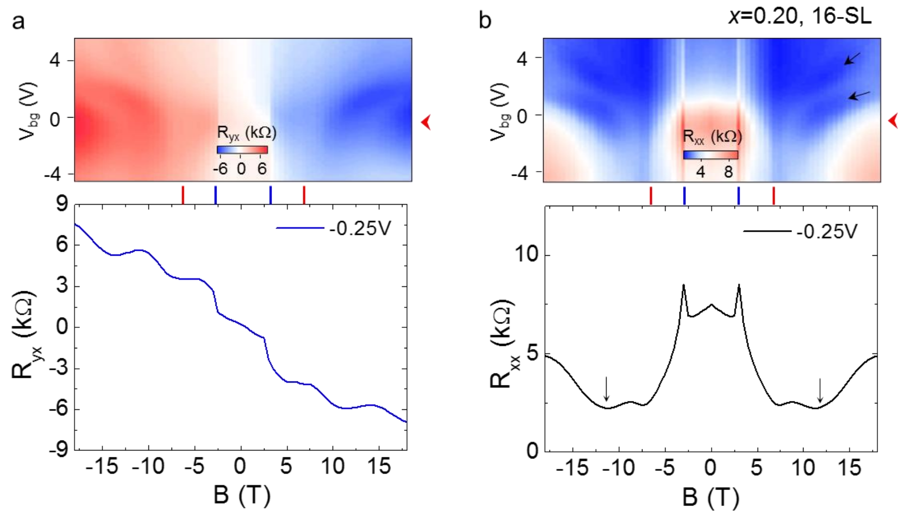

Supplementary Figure 9. Magnetotransport for 16-SL $Mn(Bi_{0.8}Sb_{0.2})_2Te_4$. Color maps of (a) R_{yx} and (b) R_{xx} as functions gate voltage and magnetic field for the 16-SL $Mn(Bi_{0.8}Sb_{0.2})_2Te_4$ measured at temperature of 1K. (bottom) The R_{yx} and R_{xx} line profiles versus magnetic field extracted from the color maps at the CNP as indicated by the black arrows. The R_{xx} and R_{yx} curves are symmetrized and antisymmetrized, respectively, with respect to the magnetic field. No C = 1 state was observed at the CNP at this film thickness for $Mn(Bi_{0.8}Sb_{0.2})_2Te_4$. The plateaus feature in R_{yx} color map and their corresponding minima in R_{xx} marked by the black arrows in (b).

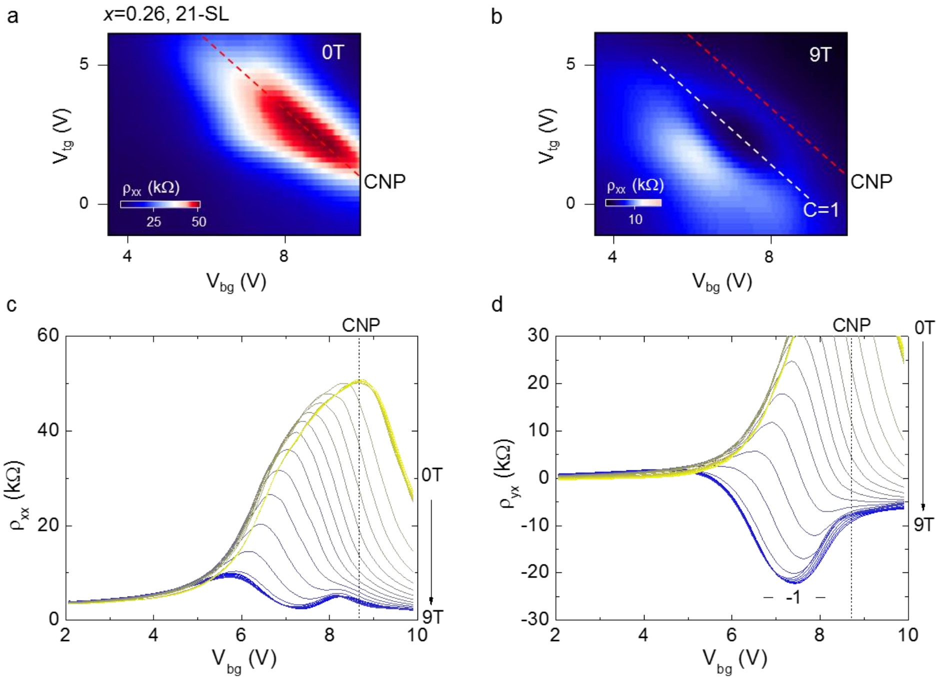

Supplementary Figure 10. Chern insulator state forming below Fermi level. Color maps of ρ_{xx} as functions of dual-gate voltages measured at magnetic field of (a) 0T and (b) 9T for the 21-SL $Mn(Bi_{0.74}Sb_{0.26})_2Te_4$ at temperature of 2K. The red and white dashed lines track the charge neutrality of ρ_{xx} maximum at 0T and the $C = 1$ state of ρ_{xx} minimum at 9T, respectively. Plots of (c) ρ_{xx} and (d) ρ_{yx} versus backgate voltage measured at different magnetic fields for the 21-SL $Mn(Bi_{0.74}Sb_{0.26})_2Te_4$. The vertical dashed lines in (c) and (d) indicate the backgate voltage corresponding to the ρ_{xx}^{CNP} at zero magnetic field.

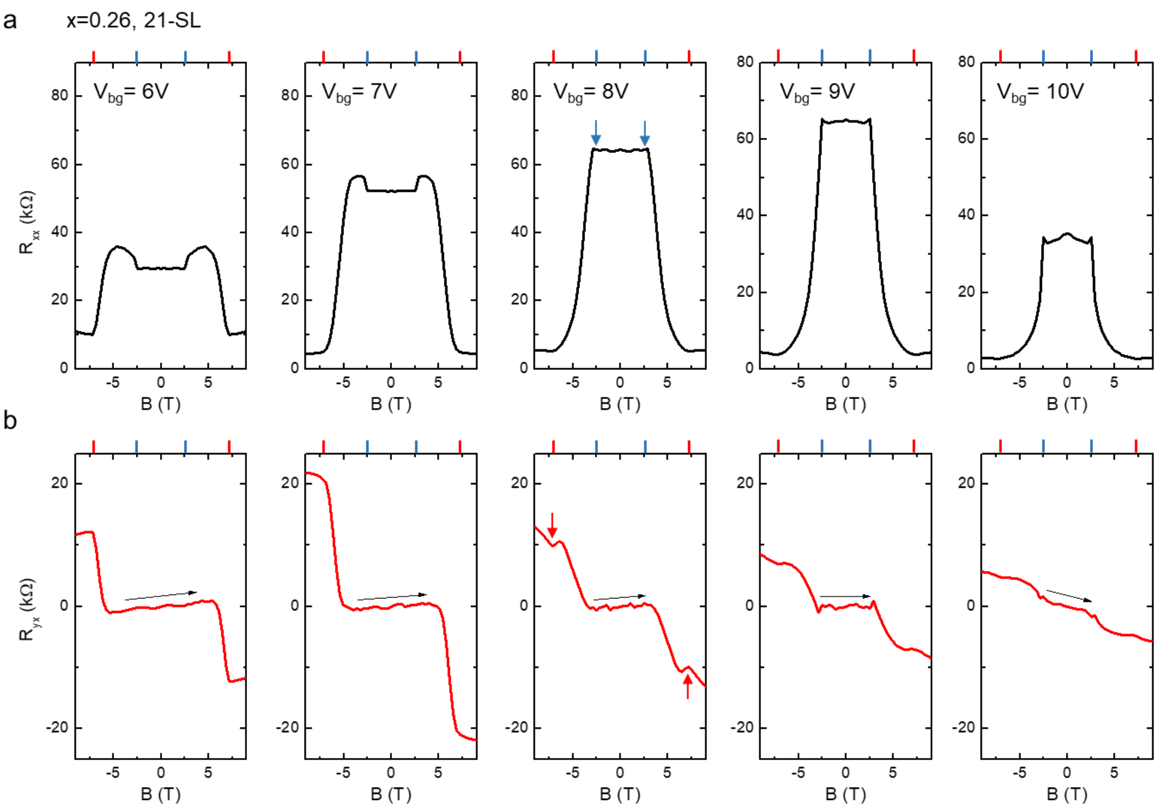

Supplementary Figure 11. Magnetotransport for 21-SL $Mn(Bi_{0.74}Sb_{0.26})_2Te_4$. The line profiles of (a) R_{xx} and (b) R_{yx} versus magnetic field for the 21-SL $Mn(Bi_{0.74}Sb_{0.26})_2Te_4$ measured at different backgate voltages. The R_{xx} and R_{yx} line profiles are symmetrized and antisymmetrized, respectively, with respect to the magnetic field. The slope of R_{yx} at low magnetic field guided by the black arrows in (b) is a consequence of the ordinary Hall effect. The positive and negative slopes correspond to the hole and electron conductions, respectively. The charge neutrality region at V_{bg} ~ +9V coincides with the R_{xx} maximum at zero magnetic field. The blue and red arrows in the panel of V_{bg} = +8V in (a) and (b) point to the spin flop and spin flip magnetic transition fields, respectively.

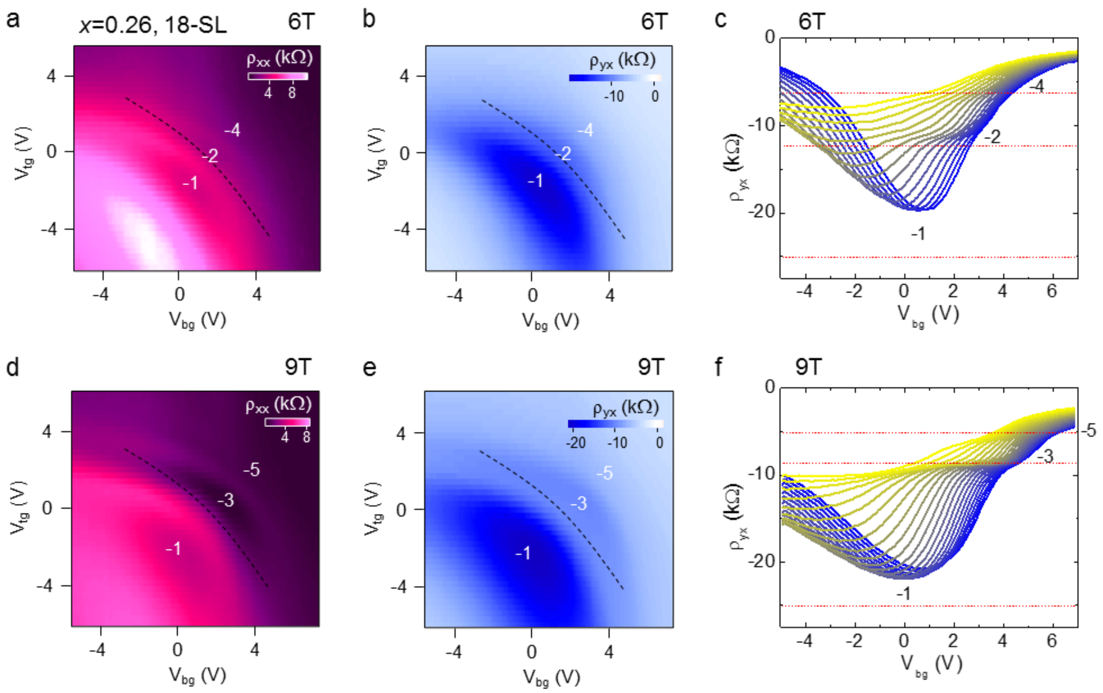

Supplementary Figure 12. Anomalous Landau levels form near the charge neutrality. Color maps of ρ_{xx} and ρ_{yx} as a function of dual-gate voltages for the 18-SL $Mn(Bi_{0.74}Sb0_{0.26})_2Te_4$ measured at magnetic field of (a, b) 6T, and (d, e) 9T at temperature of 2K. The black dashed lines in the color maps trace the CNP in dual-gating as determined from the ρ_{xx}^{CNP} at zero magnetic field. Line profiles of ρ_{yx} as a function of backgate voltage taken at different topgate voltages at magnetic field of (c) 6T, and (f) 9T. The developing Chern insulator states of the corresponded ρ_{xx} minima and ρ_{yx} plateaus are indexed in the color maps and line profiles.

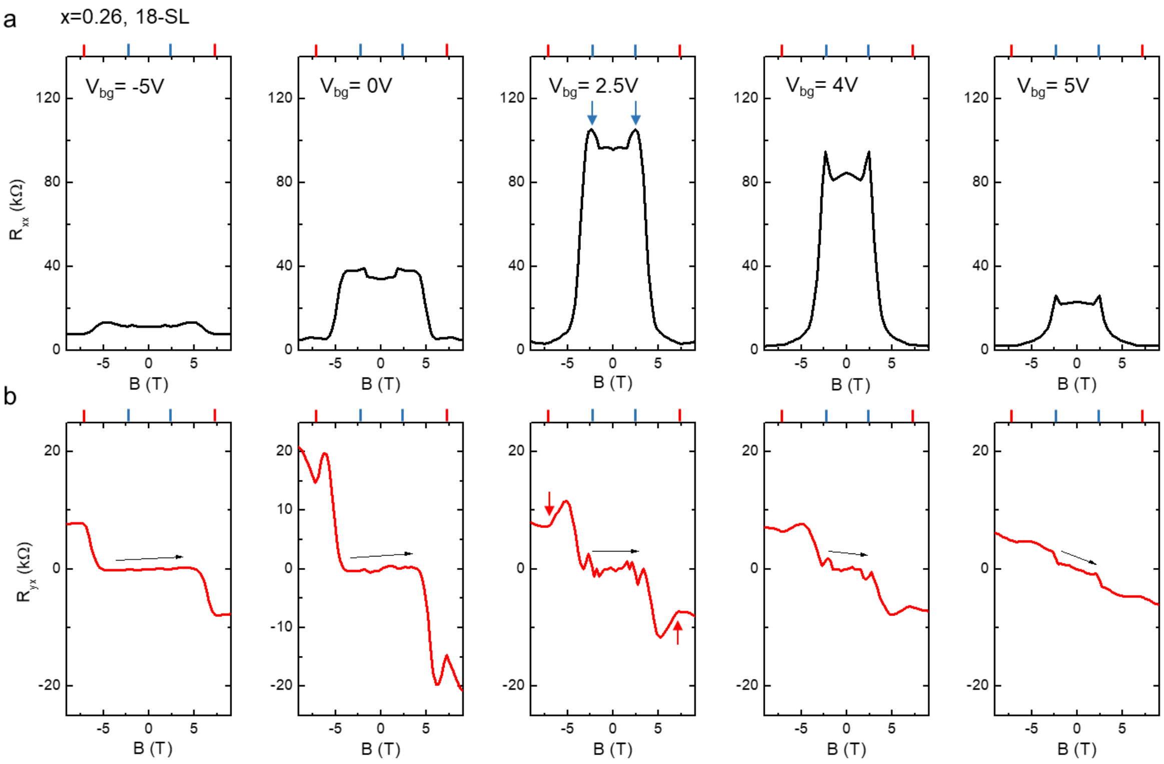

Supplementary Figure 13. Magnetotransport for 18-SL $Mn(Bi_{0.74}Sb_{0.26})_2Te_4$. The line profiles of (a) R_{xx} and (b) R_{yx} versus magnetic field for the 18-SL $Mn(Bi_{0.74}Sb_{0.26})_2Te_4$ measured at different backgate voltages. The R_{xx} and R_{yx} line profiles are symmetrized and antisymmetrized, respectively, with respect to the magnetic field. The slope of R_{yx} at low magnetic field guided by the black arrows in (b) is a consequence of the ordinary Hall effect. The positive and negative slopes correspond to the hole and electron conductions, respectively. The charge neutrality region at V_{bg} ~ +2.5V coincides with the R_{xx} maximum at zero magnetic field. The blue and red arrows in the panel of V_{bg} = +2.5V in (a) and (b) point to the spin flop and spin flip magnetic transition fields, respectively.

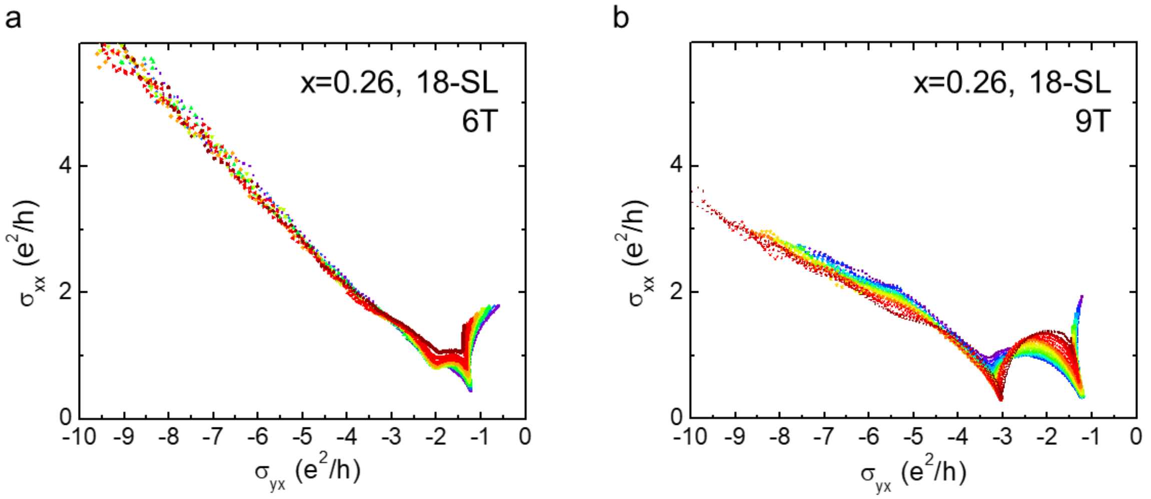

Supplementary Figure 14. Flow diagram in (σ_{xx}, σ_{xy}) parameter space. Plots of σ_{xx} versus σ_{xy} curves for the 18-SL $Mn(Bi_{0.74}Sb_{0.26})_2Te_4$ generated from a backgate voltage range of -5V to +7V at magnetic fields of (a) 6T, and (b) 9T. The σ_{xx} minima in the flow diagram corresponding to the filling factors of the Chern insulator states. The C = 1 and 2 states can be observed at magnetic field of 6T, while the C = 1 and 3 states develop at 9T. The color scales in σ_{xx} versus σ_{xy} curves correspond to the different top gate voltages controlled near the charge neutrality.

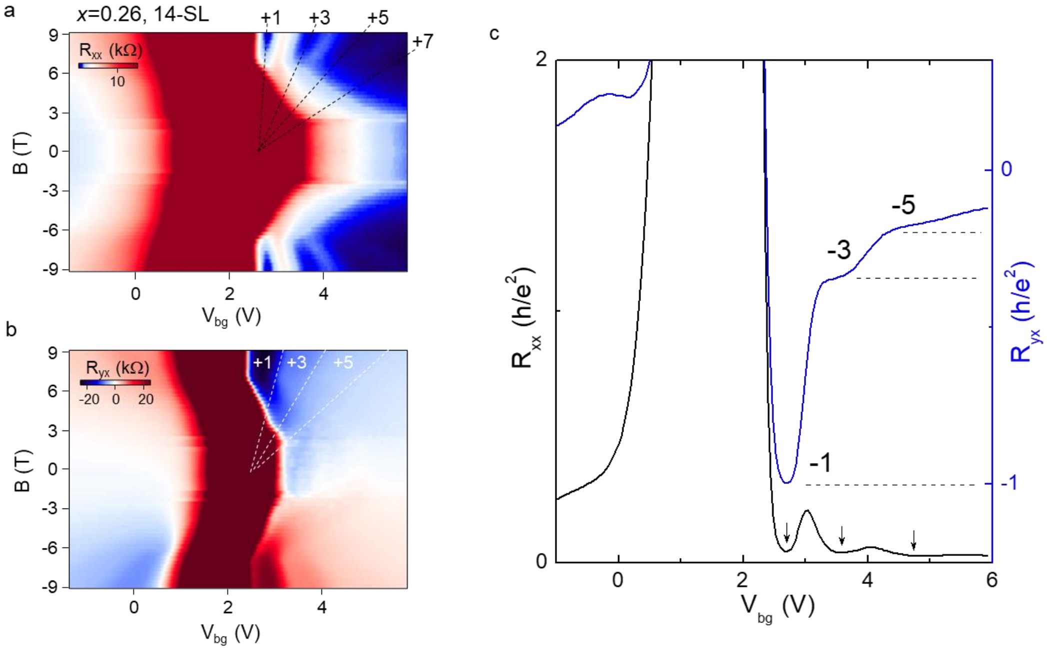

Supplementary Figure 15. Magnetotransport for 14-SL $Mn(Bi_{0.74}Sb_{0.26})_2Te_4$. Color maps of (a) R_{xx} and (b) R_{yx} as functions gate voltage and magnetic field for the 14-SL $Mn(Bi_{0.74}Sb_{0.26})_2Te_4$ measured at temperature of 2K. The Chern insulator state and Landau level filling factors developing in the ferromagnetic phase are indexed in the color maps. The black dashed lines in (a) trace the R_{xx} minima down to zero magnetic field. The white dashed lines in (b) trace the boundaries of the different filling factors. (c) Plots of R_{xx} and R_{yx} versus backgate voltage at magnetic field of 9T.

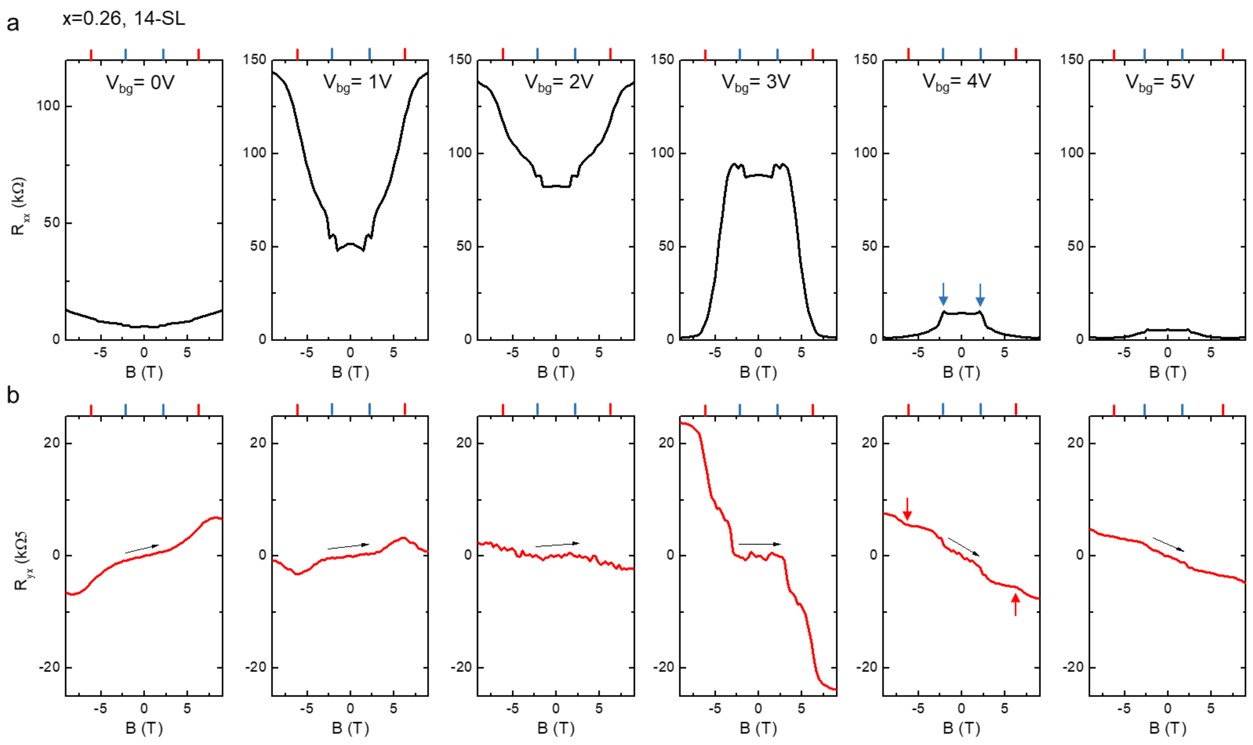

Supplementary Figure 16. Magnetotransport for 14-SL $Mn(Bi_{0.74}Sb_{0.26})_2Te_4$. The line profiles of (a) R_{xx} and (b) R_{yx} versus magnetic field for the 14-SL $Mn(Bi_{0.74}Sb_{0.26})_2Te_4$ measured at different backgate voltages. The R_{xx} and R_{yx} line profiles are symmetrized and antisymmetrized, respectively, with respect to the magnetic field. The slope of R_{yx} at low magnetic field guided by the black arrows in (b) is a consequence of the ordinary Hall effect. The positive and negative slopes correspond to the hole and electron conductions, respectively. The charge neutrality region at V_{bg} ~ +3V coincides with the R_{xx} maximum at zero magnetic field. The blue and red arrows in the panel of V_{bg} = +4V in (a) and (b) point to the spin flop and spin flip magnetic transition fields, respectively.

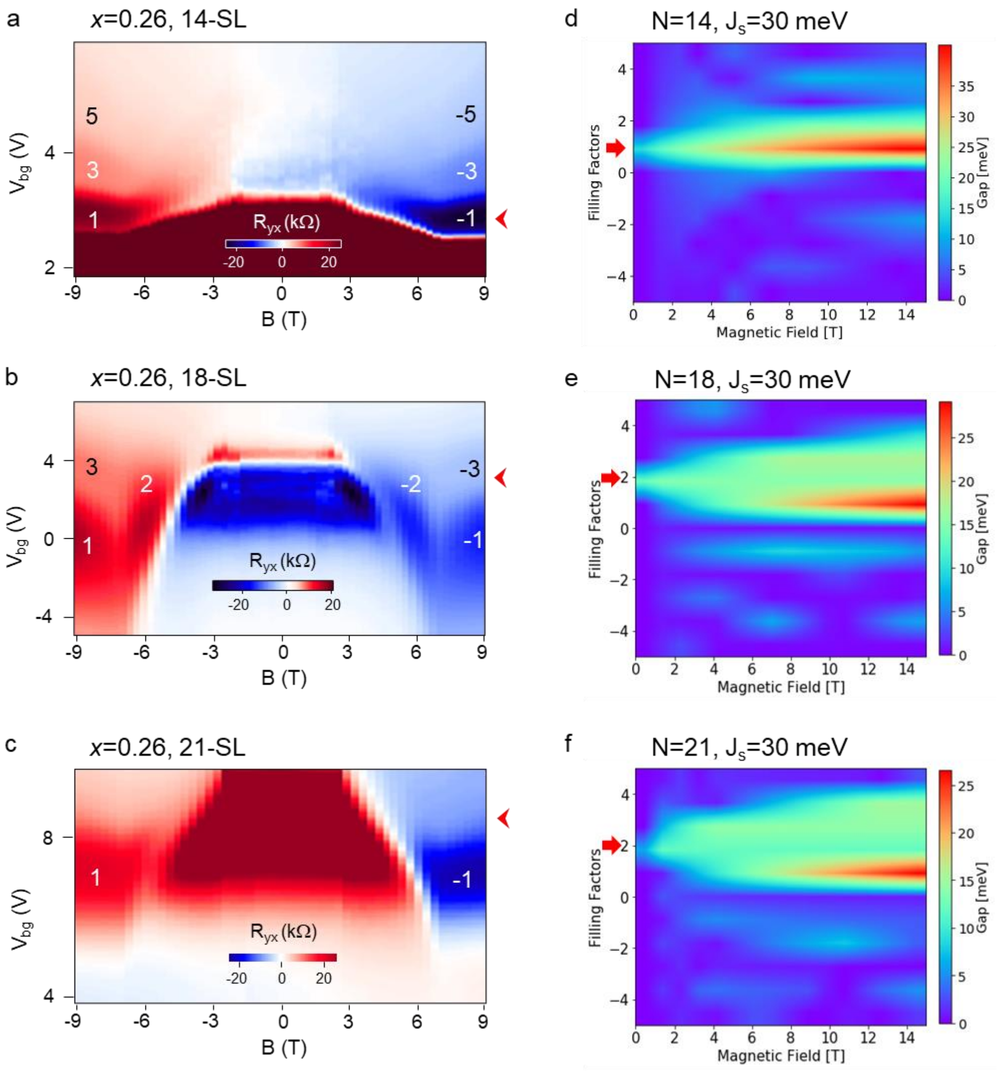

Supplementary Figure 17. Comparison between the measured and calculated LLs at different thicknesses. Color maps of ρ_{yx} as functions of magnetic field and backgate voltage for the (a) 14-SL, (b) 18-SL, and (c) 21-SL $Mn(Bi_{0.74}Sb0_{0.26})_2Te_4$. Red arrows in (a)-(c) point to the gate voltage corresponding to the ρ_{xx}^{CNP} at zero magnetic field. Contour maps of the Chern gaps as a function of magnetic field for different filling factors extracted from the anomalous and non-anomalous LLs spectra for (d) N = 14-SL, (e) N = 18-SL, and (f) N = 21-SL $Mn(Bi_{1-x}Sb_x)_2Te_4$ with $x = 0.25$. The $C = 1$ state exhibits the largest gap at a strong magnetic field for the different thicknesses. The calculations identify that the Chern number crossing point lies between 14-SLs and 18-SLs for our $Mn(Bi_{0.74}Sb0_{0.26})_2Te_4$. Red arrows in (d)-(f) indicate the Chern number determined from the spin-aligned zero magnetic field limit of the respective layer thickness.

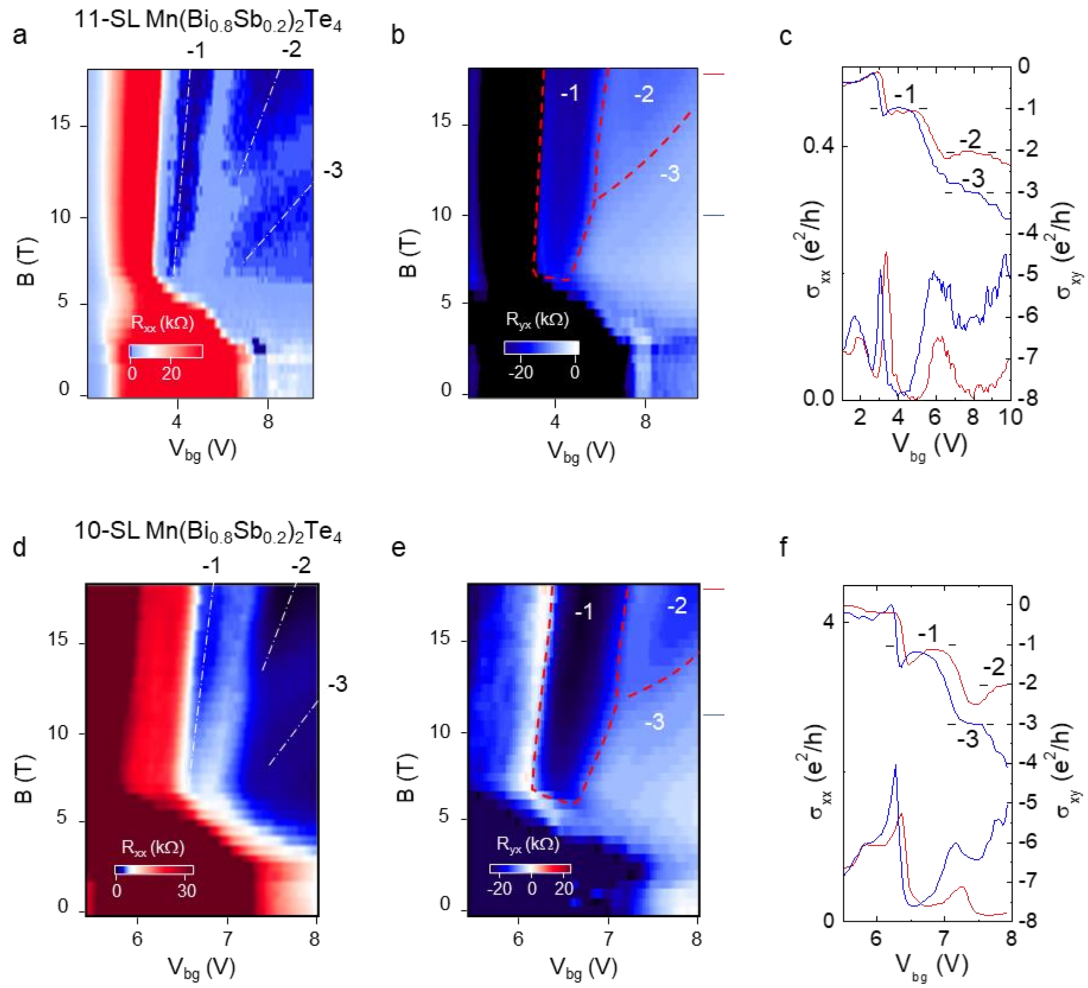

Supplementary Figure 18. Measurements at high magnetic field for $Mn(Bi_{0.8}Sb_{0.2})_2Te_4$. Color maps of R_{xx} and R_{yx} as functions gate voltage and magnetic field for the (a, b) 11-SL and (d, e) 10-SL $Mn(Bi_{0.8}Sb_{0.2})_2Te_4$. The line profiles of σ_{xx} and σ_{xy} versus gate voltage for the (c) 11-SL and (f) 10-SL $Mn(Bi_{0.8}Sb_{0.2})_2Te_4$ measured at magnetic field of 10T and 18T. The LL filling factors are indexed in the color maps and the line profiles. Similar quantization steps of C = 0, 1, and 3 states can be observed in the 11-SL and 10-SL $Mn(Bi_{0.8}Sb_{0.2})_2Te_4$ at 10T. While the additional C = 2 plateau develops at higher magnetic field of >14T for both samples. The phase boundaries traced by the red dashed lines in (b) and (e) covering the C = 1 and 2 plateaus are consistent with our theoretical picture of the anomalous LLs C = 2 state at high field as the ordinary LL bands C = 3 and above states move away from Fermi level.

Supplementary References

1. Lei, C., Chen, S. & MacDonald Allan, H. Magnetized topological insulator multilayers. *Proceedings of the National Academy of Sciences* **117**, 27224-27230 (2020).

2. Kresse, G. & Hafner, J. Ab initio molecular dynamics for liquid metals. *Physical Review B* **47**, 558-561 (1993).

3. Perdew, J. P., Burke, K. & Ernzerhof, M. Generalized Gradient Approximation Made Simple. *Physical Review Letters* **77**, 3865-3868 (1996).

4. Himmetoglu, B., Floris, A., de Gironcoli, S. & Cococcioni, M. Hubbard-corrected DFT energy functionals: The LDA+U description of correlated systems. *International Journal of Quantum Chemistry* **114**, 14-49 (2014).
